# Promoting biotechnology applications in China through effective biosafety education and communication

**DOI:** 10.1080/21645698.2025.2569930

**Published:** 2025-10-08

**Authors:** Ying Wang, Bao-Rong Lu

**Affiliations:** State Key Laboratory of Wetland Conservation and Restoration, National Observations and Research Station for Wetland Ecosystems of the Yangtze Estuary, Ministry of Education Key Laboratory for Biodiversity Science and Ecological Engineering, Institute of Eco-Chongming, School of Life Sciences, Fudan University, Shanghai, China

**Keywords:** Agriculture, biological safety, biosafety regulation, biotechnology, concept of biosafety, public awareness, teaching method

## Abstract

Biosafety represents a widespread but alarming terminology in life sciences. It was originally created for safety issues related to the possible leakage of recombinant DNAs from laboratories. However, its coverage has been greatly expanded to nearly all disciplines in life sciences. The fast development and extensive applications of biotechnology have aroused worldwide concerns over biosafety. Therefore, biosafety education becomes important for students, scientists, government staff, and the public. China has launched biosafety education and communication programs, although with many challenges. For example, it is difficult to have an appropriate definition and determined disciplines for biosafety because of its rapid evolution. Science-based knowledge and information of biosafety are still limited due to the fast change of biosafety concept, theories, and legislations. How to transform the above challenges into opportunities through effective biosafety education and communication? This article attempts to provide some thoughts and case studies for such difficulties, particularly in China.

## 1. Introduction

The current era is characterized by the fast development of biology and biotechnology with tremendous achievements in various disciplines of research, development, and applications.^1–9^ Many biology and biotechnology-related disciplines, such as molecular biology, genome sequences, gene cloning, cell and tissue culture, biological fermentation, biopharmaceuticals, genetic engineering, and synthetic biology, have made great progresses and achievements for food security and human health.^10–14^ The outcomes of these progresses and achievements are rapidly applied to various domains worldwide for commercial applications. For example, the commercialization of agrobiotechnology products has stimulated the extensive cultivation of genetically modified (GM) plant species (crops) in many countries with a total cultivation area of > 190 million hectares worldwide, according to the statistical records as of 2019.^15^ The applications of microorganism-based fermentation biotechnologies greatly promoted the development of biopharmaceutical industries.^16^ The development of sequencing-based genomic, proteomic, transcriptomic technologies, and biological systems have brought great opportunities for food security, health assurance, and elevated livelihood worldwide.^9,10^ To date, the modern biotechnologies and their widespread applications have generated great benefits in agriculture, forestry, fishery, medicine, bioindustries, and other domains. Undoubtedly, these new technologies will continue to contribute to human well-being in the future.

The rapid development and commercial applications of GM biotechnologies and their derived products in life sciences, agriculture, and medical sciences have stimulated worldwide concerns and even severe debates over the biological safety and biosafety issues associated with these advanced technologies.^17–21^ For the last few decades, biosafety represents the most widespread but controversial terminology that has considerably influenced the further development and applications of biotechnologies. This is mainly due to insufficient education and communication on biotechnologies and biosafety, which determines the understanding and support of biotechnologies by the public, including consumers. Therefore, it is very important to carry out biosafety education for the academics and the public with fundamental, easy-understanding, and correct scientific knowledge. For example, it is essential and urgent to educate university students, government employees, and people who work in the biological or biotechnological fields to understand the definition of biosafety, the historical development of the biosafety concept, the biosafety concerned research domains, the current status of national and international biosafety legislations, as well as biosafety assessment, monitoring, and management.

However, it is challenging to implement biosafety education and public communication. To date, no appropriate and consistent definition and concept of biosafety has been available, owing to the rapid advance of new biotechnologies, life sciences, and medical sciences. The constant expansion of biosafety concept to cover different disciplines also makes the stable definition difficult. In addition, it is challenging to determine the relevant domains in which biosafety is involved owing to the fast change of the disciplines linked to biosafety. Also, there are no comprehensive and authoritative biosafety textbooks available due to the fast growth of biotechnologies and their linked theories, practices, legislations, and ethical concerns. How to promote biotechnology and particularly agrobiotechnology applications through properly implementing education and communication in biosafety under the circumstances of the fast development worldwide? Correct answers will be beneficial to academics and the public for the safe and sustainable use and development of these technologies.

## 2. Historical Review of Biosafety

The emergence of biosafety problems can be traced back to the last century, in which biosafety was only closely associated with DNA recombination and transgenic biotechnology. Biosafety was originally defined as “the comprehensive measures to prevent exposure to hazardous biological agents and their spread outside laboratories” by the World Health Organization (WHO).^22^ In the late 1960s, Dr. Paul Berg, a professor of biochemistry at Stanford University in the United States, started his work on the monkey virus SV40. He attempted to use the viruses from higher animals to introduce foreign genes into eukaryotic cells as ideal carriers of prokaryotic genes. He and his colleagues tried to link a piece of DNA from a bacterium to that of the monkey virus SV40. Consequently, they managed to link different pieces of DNA from different sources together, and created the first case of recombinant DNA in the world.^23^ This major breakthrough allowed humans to alter DNA of living organisms by genetic engineering. The successful molecular and genetic modification experiments at Stanford University sparked widespread concerns and debates over their potential risks—safety problems.

At the biology conference in Cold Spring Harbor in June 1971, Dr. Berg reported his work on the transformation of a recombinant DNA into eukaryotic cells. Results of this research immediately caught the attention of biologists attended the conference, because the monkey virus SV40 was a small tumor virus that could transform cultured human cells into human tumor cells. In any case, the accidental escape and spread of these research materials into the natural environment would cause a disaster for humans because of the human-made carcinogen. Considering the potential hazards, Dr. Berg’s team stopped the research on the recombinant DNA transfection into human cells in the autumn of 1971. Since then, scientists have recognized **biological safety** or **biosafety** issues from recombinant DNA experiments.

In 1972, Dr. Boyer at the University of California of USA successfully isolated a new endonuclease from *Escherichia coli*, named *Eco*RI in his laboratory.^24^ This endonuclease could cut off DNA at a specific location, and the clipped DNA fragments could be rejoined. Therefore, this new endonuclease was called restriction endonuclease.^25^ The result of DNA cutting-and-rejoining served as a “biological knife” that could manipulate genetic materials of an organism to manipulate its genetic properties. Later, various restricted endonucleases were discovered, which allowed the manipulation of genetic materials/properties of various organisms much easier. The continued discoveries and research progress in recombinant DNAs have aroused more extensive concerns over the potential harms and risks of genetic modification and biotechnology.^26–28^

There were two important conferences during the growth of biosafety issues associated with DNA recombinants and genetically modified organisms (GMOs). The first one was the Gordon Conference held in 1973 in New Hampshire, USA, where discussions were focused on the manipulation of nucleic acids. Many scientists concerned the safety issues associated with GMOs and DNA operations, and suggested to establish a special committee to regulate the research of recombinant DNA and to formulate guidelines for the regulations. The second one was the international conference held in Asilomar, California, USA, in February 1975, where scientists formally a raised the issue of biological safety (biosafety). Since then, the biosafety concept associated with GMOs or genetically engineered organisms (GEOs) was officially proposed as an important issue that must be considered in their safe development and sustainable uses.

Nowadays, with the large scale of environmental releases and commercial applications of GMOs worldwide, biosafety is no longer the concern only by specialists and administrators, but also becomes a matter to be concerned and debated by the public. Since the formation of the biosafety terminology that was mainly focused on issues, such as the recombinant DNAs, biotechnology, and GMOs, the definition and coverage of the biosafety concept and its related contents have greatly developed and expanded, compared with its original concept. The consideration of biosafety as a concern over recombinant DNA during the Gordon Conference was then only limited to certain diseases that might affect human health in the laboratory or the local environment. However, biosafety has currently developed to cover a much wider range of safety issues that are associated with GM biotechnology and their derived GM products, in addition to the transmission of globally infectious diseases, biological invasion, biodiversity, ecological systems, and potential disasters caused by human-involved activities.^29–31^ The biosafety issues may have considerable impacts on human health, the environment, social-economic and ethical issues, and other aspects of human beings. Therefore, it is very important to implement biosafety education and communication for the national and global communities to have a better understanding of biosafety and its relevant issues.

## 3. Development of the Biosafety Concept

As the terminology implies, biological safety or biosafety is a combination of biology and safety. The terminology sounds simple, but in fact it is not so. Therefore, it is essential to really understand what is biosafety concept by its definition; how does the concept evolve with the development of its associated technologies; what are the disciplines/domains biosafety involved in; what are the relationships between biosafety and human-involved biological activities. Without a clear definition of the biosafety concept, we cannot assist people to really understand biosafety, including its natural characteristics, associated disciplines/domains, the concerned safety areas, and its relationship with scientific research, applications, and relevance to human food safety and health insurance.

The earlier concept of biosafety is related to a set of strategies, policies, legislative regulations, and procedures to ensure the safe uses of molecular biology and biotechnologies, and to prevent the possible adverse effects of these technologies on human health, the environment, biodiversity, and biological conservation.^32–34^ However, due to the fast development and expansion of biosafety associated fields, it has evolved from its original and narrow concept into a broad concept. The narrow concept of biosafety mainly focuses mainly on the safety issues associated with GM biotechnologies and their derived products. Even in the narrow concept, there are different versions of definitions for biosafety. In general, biosafety is defined as the safety issues related to human health, the environment, and biodiversity caused by the research, development, production, and practical applications of the biotechnology and its GM products.^35^

Following the definition by the United Nations (UN) Food and Agriculture Organization (FAO), biosafety was referred to as “The avoidance of risks to human health and safety, and to the conservation of the environment, as the result of the use for the research and commerce of infectious or genetically modified organisms.”^36^ Other definitions of the narrow biosafety concept were also adopted, such as the attainment of a goal to ensure that the development and uses of GM biotechnologies and their products do not have adverse impact on human health, plants, animals, genetic resources, and the environment. Though with considerable variation, the narrow definition of the biosafety concept focuses on the safety issues closely associated with GM biotechnologies and their derived products.

Following the recent Chinese Biosecurity Law (effective from April 15, 2021), the definition for biosafety refers to “the state and capability of a nation to effectively respond to the impacts, threats, and hazards from biological factors and any related risk factors, to safeguard and ensure national societal, economic, public health, and ecological security and interests”^37^ This definition emphasizes a comprehensive approach that encompasses not only food and human health of the public, but also broader national security dimensions, including socio-economic stability, biodiversity conservation, and environmental protection. Although the models of biosafety are different around the world, we used the definition of biosafety based on the Chinese Biosecurity Law in this study as the latest biosafety reference.

The fast development and expansion of biological-safety-related fields have encouraged the definition of biosafety concepts in a broader sense. The broad definition of biosafety concept includes many more aspects, such as (1) biological invasion caused by human activities and it resulted alterations and harms to native species and local ecosystems; (2) the anthropogenic changes to the environment, which threatens the existences of biodiversity; (3) the adverse or harmful effects on human health, the environment, biodiversity, and even on social life, due to the application of advanced technologies (e.g., DNA recombinant); (4) control of major new and emerging infectious diseases, animal and plant epidemics; (5) biotechnology research and applications; (6) safety management of pathogenic microbe laboratories; (7) safety management of human genetic resources and biological resources. In fact, the coverage of the biosafety concept is still expanding to more disciplines with the advances and development of biology and biotechnology.^32^ Therefore, the biosafety definition and concept may continue to expand to include all safety issues and effects closely associated with biology and human-involved biotechnologies and activities.

## 4. Main Concerned Biosafety Issues in Agrobiotechnology Domain

Since Berg’s time when the research experiment was conducted on DNA recombination in the laboratory, more and more biosafety concerns have emerged. The commercialization of the transgenic tomato “FlavrSavr” and environmental release of other GM crops restimulated the worldwide concerns over biosafety issues. Therefore, GM biotechnology per se and the extensive commercial applications of the GM or agrobiotechnology products become the primary concerns of the biosafety issues.^36,38–40^

Nowadays, there are so many diverging concerns relating to the biosafety issues. Here, we summarize the most important and influential concerns in different areas of the agrobiotechnology domain as follows.

- **Food and health**: The safety of food, feed, and human health concerns caused by the consumption of GM products.^15–18^
- **Environmental safety**: The environmental safety issues caused by the large scales of environmental release and commercial cultivation of GM products and crops in agoecosystems.^37–40^
- **Labeling and detecting system**: The system of labeling of GM or transgenic products for commercial uses, and that of detecting the presence of possible transgenes (and/or derived proteins) in agricultural products, such as GM crops.^20,41^
- **Socio-economical and ethical concerns**: The safety concerns of biotechnology and GM product induced socio-economical and ethical issues aroused by the applications of GM products and by the agrobiotechnology.^42^
- **Regulatory procedures and legislation**: The establishment and implementation of biosafety regulatory procedures, legislations, and management for GM products and agrobiotechnology-related issues^43–45^; the biosafety assessment systems in relation to the large scale of environmental releases and commercial cultivation of GM crops, as well as commercial applications of other GMOs.^46–48^
- **Public awareness**: The general public awareness and acceptance of different generations of agrobiotechnologies, and the commercial applications of GM products^49–51^; the lack of biosafety education and communication to the public and people who work in the fields of biology and biotechnologies.^34,52^

There are many concerning issues that have aroused tremendous discussions and debates over biosafety around GM biotechnologies and their derived products, but the most worrisome and focused biosafety issues are the food/feed safety and human health; the unwanted impact on the environment, ecosystems, and biodiversity; and the negative influences on social, economic, and ethical aspects. Therefore, biosafety education should also consider the following aspects as a higher priority.

First, food/feed safety and human health are the most concerning biosafety issues. Because GM agrobiotechnologies are new and still evolving, some details in the processes of GM products using agrobiotechnologies are not very clear or cannot be completely controlled, such as the transfer of a gene into the genetic background of the target organisms, which now is still randomly done. Such uncertainty may disrupt the normal DNA connection of the target organisms, causing undesired changes in metabolic processes and pleiotropic effects. Therefore, the safety and hygiene of food become the most worrisome biosafety issues for consumers and the public.

Second, the unwanted impact of GM products and their commercial applications on the environment, ecosystems, and biodiversity is another key aspect of concerning biosafety issues. Of these, the adverse effect of insect resistance gene (e.g., *Bt* gene) on non-target organisms, the environmental impact caused by transgene flow from GM crops to their wild relatives through natural hybridization, and reduction or homogenization of genetic diversity of traditional crop varieties by intensive cultivation of GM crops are typical environmental biosafety issues. All these issues should be considered for biosafety assessment of biotechnology and GM products on the environment and ecosystems.^53–56^

Third, the influences of GM crops and its extensive commercial applications on social, economic, and ethical aspects also gradually become the debated issues and concerns in the world. For example, some people consider that the adoptions of GM biotechnology and the applications of GM products have violated the natural roles and affected people’s moral and ethical views. In addition, debates are also focused on whether the GM products should be labeled (indication of a transgene or its derived compounds), and who (producers or consumers) should bear or pay the costs of the labeling processes for the GM products. Likewise, people are also anxious about whether the commercialization of GM products may increase the gaps between rich and poor (particularly, small farmers), the conflicts of economic interests, the difficulties for protecting intellectual property rights and patents. Furthermore, differences in the biosafety regulations and management (e.g., labeling of GM products), the requirement for customs inspection and quarantine, and the import and export trading policies of GMOs in different countries may create more conflicts internationally.^57^

## 5. Biosafety-Associated Research

Since its introduction, the concept of biosafety, which essentially focused on the laboratory escape and spread of recombinant DNA into the environment causing unwanted consequences, has gradually developed to cover many different disciplines related to biology. Consequently, the research areas of biosafety-related questions greatly increased from how to protect laboratory staff/technicians and prevent escape of laboratory recombinant DNAs to much more extensive fields. The focal points of biosafety research and management are also expanded widely to various directions.

With the rapid development of genetic engineering and transgenic biotechnologies, more and more GM crops and crop varieties have been successfully developed and released into the environment either for field experiments or for large-scale commercial production. All these progresses make the original biosafety concept and problems no longer limited to the laboratories and staff or local environment, which largely promoted the research of biosafety-related issues from different angles and different viewpoints. To have a better understanding of the potential problems related to agrobiotechnologies and biosafety, which is intimately related to biosafety education and communication, following biosafety-related research questions are introduced based on different areas.

- **GM biotechnology**: The biological and molecular bases of GMOs; the progresses of research and development of GM biotechnologies; the commercial applications of GMOs.
- **Risk assessment**: The theories and methodologies of potential risk assessment of agrobiotechnologies; the assessment of food/feed safety and human health associated with the commercial uses of GMOs; the assessment of ecological and environmental consequences or impacts caused by the large-scale release and commercial production of GMOs.^58^
- **Application and commercialization**: The methodologies of transboundary transfer and labeling systems for commercial application of GMOs; the liability and remedy for the large-scale release and commercial production of GMOs; the consequences and impacts associated with the socioeconomic and ethical implications from the large-scale release and commercial production of GMOs.
- **Supervisory and regulatory system**: The establishment of biosafety monitoring and management systems associated with the commercial production of GMOs; the establishment of transgene detection and tracing systems associated with GMOs; the establishment of systems linked to the international and national biosafety regulations and legislations; the establishment of an emergency regime for the safety application of GMOs.^59,60^
- **Public awareness**: Knowledge- and experience-sharing associated with the safe monitoring and management of GMOs from advanced countries; public education and participation in biosafety associated with the uses of agrobiotechnology and the commercial production of GMOs.^61,62^

The long-term and qualified biosafety research programs in different domains have generated and accumulated a great amount of scientific information and knowledge that have been published in various world’s famous academic journals, including *Nature* and *Science*. Such information and knowledge produced based on solid scientific research can be transformed into excellent teaching materials that are useful for biosafety education and communication. Such scientific information and knowledge can be refined into baseline data and guidelines for students, academies, and government employees to understand profoundly the scientific rationale of GM biotechnologies, regulatory systems, as well as the assessment, monitoring, and management of biosafety associated issues.

## 6. Biosafety Legislation and Management System

Accompanied by the fast development of biotechnology, biosafety issues have gradually attracted the attention of the international community to a great extent. To ensure the healthy development of modern biotechnologies, and at the same time, to prevent the potential adverse effects caused by the technology and its derived GM products, many countries and international organizations started to develop and implement biosafety policies, legislation, and management guidelines. These include establishing baseline data and guidelines for biosafety-associated scientific research, risk assessment, monitoring, and management of biotechnology and its derived GMO products, in addition to establishing biosafety regulations and legal documents.

Since the 1970s, the USA started to pay attention to biosafety issues, and formulated and improved the regulations on DNA recombination and transgenic biotechnology. Since 1985, Canada has developed biosafety guidelines and regulations, in addition to a relatively mature and comprehensive system for biosafety/risk assessment and management. In 1984, the European Union (EU) established the Coordinating Committee for Biosafety to coordinate the technology policies and to highlight the likelihood of biotechnology-associated biosafety problems. Obviously, the EU countries had a strict GMO management, which was very different to that of the USA. Later, other countries, such as China, India, Indonesia, Thailand, and the Philippines in Asia, and Mexico, Brazil, Chile in South America, also successively established different biosafety management mechanisms and regulations.

It was in the mid-1980s when biosafety issues concerning biotechnology and GEOs attracted the widespread international attention. In 1985, the UN Environmental Program (UNEP), the World Health Organization (WHO), UN Industrial Development Organization (UNIDO), and UN/FAO jointly formed an informal ad hoc working group on the safety of the biotechnology. These international organizations started to pay attention to the international biosafety issues. Later in 1989, the Consultative Group of International Agriculture Research (CGIAR) formed the “BIOTASK” a working group on biotechnology. Later in 1986 and 1992, the Organization for Economic Cooperation and Development (OECD) issued a series of documents on the biosafety of recombinant DNA and biotechnology, which has established a foundation for international legislation for biosafety.

It is worth pointing out that, in the 1990s, the development of an important international document particularly connected with the biosafety issues was proposed under the framework of the Convention on Biological Diversity (CBD), where the safety of biotechnology was indicated. In 1995, the CBD Conference of Parties decided to draft a biosafety protocol. After five years of hard work and negotiations, the full text document of the Cartagena Protocol on Biosafety was completed on January 29, 2000, and opened for signing in Nairobi, Kenya during May 15 to 26 in the same year. The Chinese government signed and ratified the Cartagena Protocol in August 2000 and in September 2005, respectively. The Cartagena Protocol on Biosafety finally took effect on 11 September 2003 after signed and ratified by > 150 countries. Since then, the Cartagena Protocol on Biosafety has become an internationally authoritative document used for legislation and management of biosafety issues. The initiation and development of the national and international regulatory and legal documents have served as the foundation of biosafety assessment, monitoring, and management.

To date, more comprehensive systems regarding the biosafety legislations are established nationally or internationally to guarantee the safe and healthy research and development of biotechnologies. In addition, the safe and sustainable applications of biotechnologies and GMO products are taken into conscientious considerations. However, knowledge and information related to legislations, based on which the research and development of biotechnologies, biosafety assessment, and management are implemented, have not been well spread and communicated. Therefore, education and communication of biosafety related to legislations should also be deployed to catch up the strides of biotechnology development.

## 7. Challenge of Biosafety Education: A Case Study in China

China is one of the largest countries in the world in terms of its human population. Therefore, to meet the great demands for foods and to resolve the national food security problems is always the first-priority efforts and goals for Chinese government. In fact, China has been successful in feeding > 20% of the world’s population, based only on < 8% of the world’s farmlands. Such a great achievement to feed a huge population with sufficient food supplies is already a miracle for a country. In this regard, the Chinese government fully supports and promotes the research, development, and commercial applications of the high-and-new technologies, including biotechnologies to elevate the living standard of Chinese people, in addition to meeting the challenges of the long-term and stable food demands for the increasing population.^32,38^

China started the research and development of GM biotechnology relatively later than the industrial countries such as the USA and Canada. However, the research and development of GM biotechnology progressed rapidly in China, because of the prioritized efforts and goals by the government who has profoundly recognized the importance of GM biotechnologies and invested tremendously in developing GM biotechnologies. In the 1990s, many research programs and foundations such as, the well-known National Key Science and Technology Program (863 Program), the National Key Basic Research and Development Program (973 Program), and the Natural Science Foundation of China (NSFC) strongly supported the bioscience- and biotechnology-associated researches and development. Consequently, many research institutes, universities, national and provincial key laboratories, and other research initiatives were actively involved in the research and development of GM biotechnology.^32,38^

In addition, life sciences and biotechnologies are listed as prioritized high-and-new technologies and industries for the research and development. Particularly, the outline of the National Program for Medium to Long-term Development of Science and Technology listed the research and development of GM crop varieties, livestock, and poultry breeds as one of the 16 key programs in 2008. In the same year, the Chinese government lounged the “Major Project to Breed New Varieties of Genetically Modified Organisms” with the investment of an equivalent value of ~3.5 billion US dollars. Altogether, these demonstrated the strong well of China in research, development, and applications of the new GM biotechnologies for food security and human health. Undoubtedly, with such desires and ambition, China has made a great progress in the research and development of transgenic biotechnology.^32,38^

Scientific communication and research of biosafety issues in China started even later than those of GM biotechnology development. In general, basic information and knowledge concerning the GM biotechnology and biosafety issues were extremely limited until the beginning of 2000s. Biosafety education and its associated knowledge was much lagging behind the GM biotechnologies. Only a few universities (e.g., Fudan University and Minzu University of China) had biosafety syllabus.^35^ Textbooks relevant to biosafety courses and teaching are also very scarce. In addition, limited public understanding of GM biotechnology and its related biosafety issues aroused great concerns over the food and health safety, and impacts on the environment and biodiversity. In addition, the lack of knowledge on the labeling of GM products, detection of transgenes, and on the relevant regulation and legislative systems greatly hindered the development and applications of GM biotechnology and its products. Ignorance of GM biotechnology and lack of biosafety education have resulted in confusion, conflict, fear, and resistance to GM products. Consequently, the commercial applications of GM biotechnologies and their products, particularly those with Chinese intellectual property rights, become very difficult in China.^38^

For appropriate assessment and management of agrobiotechnologies and GM products for commercialization, the Chinese government has gradually established comprehensive legislative systems. In 1990, a series of biosafety standards and measures were issued, including the “Quality Control Standards for Genetically Engineered Products.” In 1993, the State Science and Technology Commission (now the Ministry of Science and Technology) issued the “Measures for the Safety Management of Genetic Engineering,” which was the first system for the management of GMOs in China. In 1996, the Ministry of Agriculture (now the Ministry of Agriculture and Rural Affairs) implemented the “Measures for the Safety Management of Agricultural Biological Genetic Engineering.” These measures are specifically and highly targeted on a wide range of areas with relatively clear information for the safety assessment of GMOs.

In 2001, the State Council issued the “Regulations on the Safety Management of Agricultural Genetically Modified Organisms (RSMAGMO),” which was the highest level of legislation for the biosafety management of GMOs. Soon after that, the Ministry of Agriculture issued a series of regulations and technical standards to cope with RSMAGMO for the management of agricultural GMOs, including their safety assessment, imports, identification, labeling, and general administrations. In April 2021, the Chinese government issued the “Biosafety Law of the People’s Republic of China,” in which the concept and contents of biosafety are largely expanded with the coverage of many fields.^37^ According to the Chinese Biosafety Law, the contents of biosafety are greatly expanded from biotechnologies and GMOs to seven different disciplines: (1) biosafety risk prevention and control system; (2) prevention and control of major new or sudden outbreaks of infectious diseases and epidemics in animals and plants; (3) safety of biotechnology research, development, and applications; (4) bio-laboratory biosafety of pathogenic microorganisms; (5) safety of human genetic resources and biological resources; (6) prevention of the threats of bioterrorism and biological weapons; (7) the capacity buildings for biosafety. So far, the coverage of the Biosafety Law is the most authoritative and up-dated legislation of biosafety in China.

Currently, the most prominent challenges for biosafety education can be listed as follows. First, the definition of biosafety is continuously evolving and the coverage and contents of biosafety are constantly expanding to broader disciplines, from only dealing with GM biotechnologies although the situation varies in different countries or regions. Second, coped with such alterations, the requirements for the biosafety evaluation, monitoring, management, and the regulation procedures, as well as the associated legislations are also changing at different times. Third, there are huge requirements of professionals for biosafety assessment and management of new-generation agrobiotechnologies and GMOs by human society in the future. Fourth, the requirement from the public to strongly support the research and development of the agrobiotechnologies and GMOs. How should we determine the appropriate biosafety concept and contents in biosafety education under complex situations? Therefore, it is urgently necessary to establish an appropriate and scientifically sound biosafety education system for students, academies, government staff, and the public for better understanding of biosafety, in order to guarantee and promote applications of agrobiotechnologies and their products for food security and human health in China and other countries.

## 8. Opportunities and Future Perspectives

Only after a few decades the modern biotechnology, which was initially developed based on DNA recombination and genetic modification, has had tremendous progresses and achievements globally.^1–9^ Modern biotechnology is still developing quickly, which has affected different disciplines in life sciences and other related fields in terms of their research, development, and applications. Winning the huge benefits, many countries including both industrial and developing countries have listed the research and development of biotechnology as one of the key policies for further economic development and takeoff in their programmed agenda. The research and development of biotechnology and commercial application of GM products also become the important strategies of many countries for the international competition in the future, particularly for developing countries. In addition, new generations of biotechnology and molecular biology, such as advanced gene editing and next generation DNA sequencing, are invented and developed quickly, which will have a great impact on the livelihoods of global populations. Undoubtedly, the new generation biotechnologies and GM-associated industries will rapidly become the rising pillars to resolve the food security problems, as well as to meet future demands for medicine and human health in the world.^9–14^ Diverse types of biotechnology derived or GM products will appear on the tables of ordinary families and become everyday foods for many people.

However, the key question is that the public who resist the development and applications of the biotechnology and its products do not even know what is the technology, what benefit and potential adverse effects this technology can bring to people’s livelihood and well-being. They do not have basic knowledge and ideas that the technology can effectively improve crop varieties and animal breeds, and can help to resolve the world's food security problems. All the problems are caused by insufficient education and communication on biosafety and its associated biological and biotechnological development.^35^ Therefore, it is very important to have biosafety education and communication with systematic science-based knowledge. Such education and communication can help the public to avoid blind fears or even resistance to biotechnology and its products without any reasons.

For the strategic design of a better biosafety education and communication system, we should learn from the available experiences of biosafety education in China and other developed countries. The comparison of biosafety education frameworks and models from China, the United States of America (USA), and European Union (EU) will provide valuable insights and references for a more strategic education system (Table 1).

**Table 1.** Comparison of biosafety education models in China, the United States of America (USA), and the European Union (EU).

Content	China	USA	EU
Legislation Basis	Biosecurity Law of the People’s Republic of China (2021).	Public Health Security and Bioterrorism Preparedness and Response Act (2002).	Animal Health Law, Directive on the Deliberate Release of GMOs (2016).
Education objectives	Enhance public biosafety awareness to safeguard national security; educate professionals for pandemic response.	Strengthen laboratory protocols and counterterrorism capabilities; foster systemic thinking and problem-solving skills.	Ensure animal health and food safety, prevent cross-border diseases; emphasize public participation and risk prevention.
Implementation entities	Government-led, with collaboration from research institutions, medical facilities, and universities.	Federal and state government division of labor; multi-stakeholder collaboration.	EU-wide legislation enforced by member states; joint participation by research institutions and public organizations.
Core Methods	1. Curriculum integration: Biosafety modules in school biology courses, mandatory university courses. 2. Practical drills: Laboratory safety exercises, clinical training. 3. Technology empowerment: VR virtual laboratories, short-video science outreach.	1. Laboratory classification: BSL-1 to BSL-4 risk controls with mandatory safety training. 2. EIC (Environment as an Integrating Context) model: Environment as a learning context. 3. Systematic teaching materials: textbooks with safety icons and protocols (e.g., handling corrosive substances).	1. Risk assessment: Strict GMO safety assessments with tiered management. 2. Public outreach: Community campaigns, labeling systems. 3. Cross-border coordination: Joint animal disease monitoring among member states.
Key focus areas	Agricultural biosafety (e.g., vector insect control), major infectious disease response.	Synthetic gene technology oversight (e.g., DNA sequence screening, GMOs).	GMOs regulation, prevention of cross-border plant and animal disease.

Obviously, the available biosafety education frameworks and models in China, the United States of America, and the European Union are different. It is necessary to highlight the key points of the education models. China has a national safety-oriented public education model based on the modules in school biology courses, university “theory-practice-clinical” training chains. The key distinction is aligning biosafety education with public health and agricultural security under the national strategy.^37,63^ The USA has a technology-practice-focused laboratory safety model based on tiered biosafety management, field-based learning (e.g., nature reserves), and textbook safety design systems.^64,65^ EU has a precautionary principle-driven legislative model, emphasizing dual oversight via horizontal (e.g., GMO directives) and product-specific (e.g., GMO food labels) regulations.^66,67^ In summary, China has a national security-centered, government-led public education with focus on agriculture and pandemics; the USA implements a laboratory safety–driven model, combining technical practice and counterterrorism with rigorous protocols; whereas the EU emphasizes a precautionary principle-based systemic legislation for food safety and cross-border disease control.

Based on the existing challenges in China and available experiences in the world, here we propose the following strategies on biosafety education and public communication for university students, academies, and the public. These strategies can be referred to and used in China and in other countries.

- **To integrate biosafety into curricula**: Embed biosafety principles, concept, and the legislation systems, e.g., The Biosafety Law of the People’s Republic of China, into life sciences, biotechnology, and related courses, emphasizing biosafety in the laboratories, fields, and ecosystems, and respecting DNA recombination and pathogen research protocols.
- **To increase hands-on laboratory training**: Conduct mandatory biosafety workshops and trainings for students to correctly handle molecular experiments and high-risk pathogens, focusing on contamination prevention and emergency responses.
- **To enhance case-based learning**: Use real-world scenarios (e.g., environmental release of GMOs and COVID-19 outbreaks) to teach risk assessment and ethical responsibilities in biotechnology research.
- **To have regular professional training**: Organize annual training programs of biosafety management and technical certification for laboratory personnel, covering regulatory compliance and advanced containment measures.
- **To encourage interdisciplinary collaboration**: Establish joint platforms between universities, research institutes, and health agencies to share best practices in molecular experiments, pathogen research, and GMO safety.
- **To establish expert-led workshops**: Invite experts university professors to deliver lectures on emerging biothreats and innovative biosafety, bridging research gaps with policy needs.
- **To emphasize national security campaigns**: Leverage a National Security Education Day to promote biosecurity and biosafety awareness via media, highlighting the Biosafety Law and its role in preventing relevant problems.
- **To establish accessible network for science-based biosafety communication**: Use effective formats, e.g., public lectures and TV programs by scientists to explain biosafety of GMO-related issues, benefits brought by biotechnologies, infectious diseases control, in layman’s terms and language.
- **To have community engagement**: Partner with customs and local health departments to run exhibitions on border biosecurity and biosafety, educating citizens on preventing such problems as invasive species and disease spread.

In conclusion, human’s experiences and scientific knowledge of new-generation biotechnology and its associated biosafety issues are quickly increased and accumulated because of the fast development of the biotechnology and applications of its derived products. In order to promote the sustainable development and wide applications of biotechnologies for food security, human health, and biodiversity, better understanding of biosafety and its associated scientific issues is essential and important. However, there are many challenges in biosafety education and communication, because of the constant change and adjustment of biosafety definition, concept, and content, along with the fast development of new biotechnologies and socio-economic requirements for biosafety assessment and regulations. Nevertheless, biosafety education and communication with appropriate information and science-based knowledge will help to greatly eliminate public fears about biotechnologies and GMOs as well as the associated biosafety issues. Such education and communication will establish a stable and harmonious global community in terms of promoting applications of biotechnologies to their products.
